# Workplace bullying in intensive care units: intern nurses’ experiences and responses

**DOI:** 10.1186/s12912-026-04599-8

**Published:** 2026-04-13

**Authors:** Rawia Gamil, Ahmed Mohamed Ismail Zerea, Fatma Refaat Ahmed, Muhammad Arsyad Subu, Heba Mustafa

**Affiliations:** 1 Department of Critical Care and Emergency Nursing, Faculty of Nursing, Alexandria University, Alexandria, Egypt; 2 Department of Emergency and Intensive Care Nursing, Northern Border University, Arar, Saudi Arabia; 3 Department of Nursing, College of Health Sciences, University of Sharjah, Sharjah, UAE

**Keywords:** Bullying, Intern nurses, ICU, Mixed methods, Workplace violence

## Abstract

**Background:**

This study explores how bullying negatively affects intern nurses’ assertiveness, performance, and the quality of patient care. It emphasizes the need for supportive, respectful clinical environments to enhance interns’ confidence, safety, and professional development.

**Aim of the study:**

This study aims to explore the intern nurses’ responses to bullying in the ICU.

**Methods:**

A convergent mixed-methods design was used to gain a comprehensive understanding of bullying among intern nurses in the ICUs of Alexandria University Hospitals in Egypt. questionnaire. Of 610 invited interns, 200 completed the questionnaire, and 29 provided narrative responses; a pilot study with 10% of the sample tested feasibility before data collection. The quantitative data were collected using the Negative Act Questionnaire (NAQ-R), and the qualitative data were collected using three questions developed by the researcher.

**Results:**

The study revealed that over half of the intern nurses (53%) experienced bullying and about half witnessed it (47%), mainly due to their limited experience, unfamiliarity with ICU routines, and hierarchical workplace stress. The overall mean bullying score was (2.17 ± 1.00), with work-related bullying observed to have the highest mean score at (2.37 ± 1.17), followed by physically intimidating behaviors (2.35 ± 1.25), and person-oriented bullying (2.01 ± 0.97). In addition, there were statistically significant associations between the three dimensions of bullying experience and intern nurses’ age (*p* < 0.001, *p* < 0.001, and *p* = 0.002). Professional and social vulnerability to workplace bullying, the emotional reactions to bullying and its effect on intern nurses’ professional conduct, coping with bullying, and intern nurses’ explicit messages and suggestions were the four main identified themes from the qualitative analysis.

**Conclusions:**

The study revealed a high prevalence of workplace bullying among intern nurses, mainly work-related and physically intimidating, emphasizing the need for institutional interventions and support systems. It calls for pre-internship training on coping strategies, faculty mentorship programs, and clear, confidential reporting procedures to prevent and address bullying effectively.

## Introduction

The internship year is designed to enhance intern nurses’ clinical competence by engaging them in direct patient care under the supervision of a preceptor. Unlike the first four academic years, during which nursing interns primarily observe registered nurses and occasionally assist with basic procedures under the close supervision of the clinical tutors, the internship year immerses them in real-world clinical practice. This transitional period, while essential for preparing intern nurses to assume full professional responsibilities, is often challenging due to intern nurses’ heightened vulnerability to workplace harassment and bullying. This would be more frequent among nurses who are working in a dynamic work environment like intensive care units (ICUs) [1–3].

Bullying in work environments can occur between colleagues at different hierarchical groups and is more prevalent among vulnerable individuals including intern nurses, new hires, or even experienced staff. Bullying has emerged as a significant concern commonly reported by intern nurses, and these behaviors perpetrated by the bullies are mainly from individuals who have a higher level of authority. Bullying is often used interchangeably with workplace violence [4] and is defined as repeated aggressive behavior intended to harm someone, typically intended to assert the aggressor’s dominance over the victim. Bullying can take many forms, including verbal, social, and physical acts, such as hitting or other violent actions [5–7]. 

Intern nurses are highly vulnerable to bullying due to their younger age, limited clinical and life experience, and low position within the organizational hierarchy. In ICU settings, this vulnerability may be intensified by high workloads, critical decision-making demands, and frequent interactions with multidisciplinary teams, patients, and families. Intern nurses may be exposed to bullying from multiple sources, including peers, senior nurses, physicians, patients, and patients’ relatives. Such exposure has been shown to negatively influence intern nurses’ performance, assertiveness, and confidence, potentially increasing the risk of errors and decreasing the quality of nursing care [3, 5, 9]. 

The consequences of bullying are predominantly negative and might impact the well-being of individuals, leading to emotional distress, anxiety, depression, and decreased job satisfaction. In addition, bullying undermines teamwork, disrupts communication, compromises patient care, and reduces overall workplace morale. Despite the deleterious effects of workplace bullying, fewer studies have examined how intern nurses cope with these behaviors, particularly in high-acuity settings such as ICUs [5, 8]. 

Coping strategies reflect the intern nurses’ efforts to withstand and survive adverse experiences, may include emotional regulation (e.g., internalization, positive self- talk, and mindfulness), seeking social support, avoidance, problem-focused strategies, or maintaining professionalism and a focus on patient care. Some intern nurses report developing resilience, stress-management skills, or heightened awareness of workplace dynamics when confronted with bullying, thereby avoiding repeated exposure to such experiences [9–10]. Moreover, exposure to bullying may increase intern nurses’ awareness of power imbalances of some health care providers, motivating some to act and advocate for respectful communication, peer support, and anti-bullying procedures. Such insights may influence their professional values and commitment to fostering healthier work environments [8, 11–13].

Despite the growing body of research on workplace bullying in nursing, there is a gap in studying intern nurses’ experience of bullying and their coping strategies, particularly in highly stressful workplace environments such as the ICUs. Most existing studies focus on prevalence and negative outcomes, with limited attention to how intern nurses manage, adapt, or respond to bullying during this critical stage of professional development [21–23]. Addressing this gap is important to implement suitable interventions targeted at these aggressive behaviors and to promote positive clinical learning environments. Therefore, this study aims to explore the responses of intern nurses when exposed to workplace bullying.

### Study aim

The aim of this study is to explore intern nurses’ responses to bullying in the ICU. This aim is achieved through five research objectives:

1. Describe the frequency of exposure to/witnessing bullying among intern nurses.
2. Identify the most prevalent type of bullying experienced by the intern nurses.
3. Explore the factors associated with intern nurses’ exposure to bullying.
4. Discuss the impact of exposure to bullying as perceived by the intern nurses.
5. Determine the strategies the intern nurses adopt to deal with bullying.

### Study design, setting, and participants

A convergent mixed-methods research design Teddlie, (2007) [14] was employed in this study, given its novelty; the combination of quantitative and qualitative data provides a deeper understanding of the issue being researched. This study was conducted at the intensive care units of Alexandria University Hospitals in Alexandria, Egypt. The ICUs studied admit patients with various disorders at the acute stage of their illnesses. Intern nurses who had completed 12 months of internship training were recruited conveniently to participate in this study. 610 were invited to participate in the study through the official academic email with information explaining the aim of the study; 200 intern nurses completed the study questionnaire and were then asked to answer the narrative questions until achieving data saturation from 29 participants who agreed to answer the narrative questions. A pilot study was conducted with 10% of the study sample to assess feasibility; interns who participated in the pilot were excluded from the final study sample.

### Measurement

Data was collected from January 2025 to February 2025. **The quantitative data (objective 1**,** 2) were** collected using an online questionnaire, which comprised two sections. Section  1: the demographic and work-related data, such as the intern nurse’s age, sex, marital status, and working unit; Section  2: the Negative Acts Questionnaire-Revised (NAQ-R), which was adopted from Einarsen et al. (2009) [15]. It is used to determine exposure to workplace bullying and contains items for each of the three distinguished forms of bullying: person-oriented, work-related, and social exclusion. It consists of a 22-item, person-oriented bullying, including social isolation, which is measured with 12 items, 7 items point to work-related bullying items, and physically intimidating bullying is measured with three items. There are five possible answers (Never; 1 Now and then (occasionally); 2 Monthly; 3 Weekly; 4 Daily; 5). After scoring each item, the results are calculated and divided by 22 to get the final score range (1–5). This scale is valid and has a reliability coefficient for the total and subscale scores of the NAQ-R, ranging from 0.63 to 0.90.

The **qualitative data (objective 3–5)** collected using three narrative questions developed by the researchers to provide more details about bullying that are not included in the questionnaire and gave the intern nurses the opportunity to express, using their own words, their response to bullying and describe the potential effect on their professional development. These questions were: (1) Can you describe the circumstances that contributed to the bullying you faced/witnessed? (2) How did you handle bullying when it happened? (3) How do you feel the bullying has influenced you? Thematic analysis method was used to identify, analyze, and report themes within the collected qualitative data. An inductive coding strategy was used, and the codes and themes were obtained directly from data reported by the study participants. Two researchers were responsible for the coding process; in case of disagreement or discrepancy, a third researcher made the final decision to ensure reliability and consistency in theme development.

### Data collection

The online questionnaire was sent to eligible intern nurses via an official university email. Those who agreed to participate in the study were asked to sign an informed consent. An appointment was arranged with the participants if they had any questions about the questionnaire, they were informed to contact the researchers. After completing the questionnaire, the participants were asked to answer three narrative questions developed by the researchers. To address the possibility of non-response bias, where respondents who participated in the study may differ from those who didn’t, the questionnaire remained active for a month, during which repeated invitations were sent to all eligible participants to encourage participation and increase the response rate.

The online questionnaire remains active for one month to improve the response rate, with periodic invitations to participate in the study as reminders for participants who may have missed the initial invitation email.

### Ethical considerations

This study has been conducted according to the Declaration of Helsinki in 1964. Before beginning this study, approval was obtained from the Research Ethics Committee of the Faculty of Nursing at Alexandria University, Egypt (approval number: 20-2-327). Informed consent was obtained from all participants prior to their participation in the study. Participation was voluntary, and participants were informed of their right to withdraw at any time without any consequences. Anonymity and confidentiality of all collected data were strictly maintained.5

### Trustworthiness

The trustworthiness of the qualitative component in this study was assessed using credibility, transferability, dependability, and confirmability [41–43]. The opinions of experts regarding data extraction, analysis, coding, and classification were taken into consideration. We described the study phases in detail, including the study settings, ensure transferability and enable readers to review the procedure. To ensure the intended meanings were conveyed accurately, two intensive care unit nurses reviewed the transcripts. Comprehensive drafts of the study phases were created to improve dependability and confirmability. This allowed authors to track the data and comprehend one another’s interpretations. The authors also held biweekly discussions during the data collection period to assess the coherence of their assessments and perceptions. To ensure reflexivity, the authors’ identities were kept secret from every study participant.

### Rigor

The trustworthiness was linked to the steps of qualitative analysis to enhance rigor as follows: credibility was maintained through employing two researchers for coding and discussion to double check and ensure accurate interpretation of data; dependability was maintained through documenting the steps of data analysis and coding decisions; confirmability was maintained through employing a third researcher to discuss and take final decision in case of coding discrepancies; and transferability was ensured by providing detailed description of qualitative data obtained from participants and the study context.

## Results

Table 1 presents the demographics and work-related data for the respondents. A total of 200 intern nurses returned the online questionnaire. Of these participants, 29 answered the narrative written questions after completing the questionnaire. Most of the respondents were female (80%) and predominantly aged 22 years (43%), with a mean age of 24.01 ± 3.19 years. Most of the participants were single (75.5%). Regarding their placement, the vast majority received internship training in General ICUs (85%), while only 6% and 9% were in emergency and surgical ICUs, respectively.

**Table 1:** Demographics and work-related data of the respondents studied. *N* = 200

Variable	Categories	No.	%
Gender	Female	160	80%
	Male	40	20%
Age (yrs)	22	86	43%
	23	42	21%
	24	22	11%
	25	18	9%
	26–29	14	7%
	30+	18	9%
	Range (Min - Max)	22–41	
	Mean ± SD	24.01 ± 3.19	
Marital Status	Single	151	75.5%
	Married	45	22.5%
	Others	4	2%
Work Unit	ER ICU	12	6%
	General ICU	170	85%
	Surgical ICU	18	9%

Table 2 shows the experience of bullying among the studied respondents. More than half of the respondents (53%) reported being directly subjected to bullying during their internship training, while 47% had witnessed bullying incidents involving others. These findings highlight the widespread presence of bullying, either directly or indirectly.

**Table 2:** Experience of bullying among studied respondents, *N* = 200

Categories	No.	%
Witnessed bullying	94	47%
Subjected to bullying	106	53%

Table 3 provides an overview of the negative acts questionnaire-revised (NAQ-R) of the respondents studied. The analysis of the Negative Acts Questionnaire-Revised (NAQ-R) responses among intern nurses revealed that, across multiple dimensions, the overall mean bullying score was 2.17 ± 1.00, indicating that while some individuals experience minimal bullying, others report frequent negative acts. When examining subscales, work-related bullying was observed to have the highest mean score at 2.37 ± 1.17, followed by physically intimidating behaviors at 2.35 ± 1.25, and person-oriented bullying at 2.01 ± 0.97.

**Table 3:** Negative Acts Questionnaire-Revised (NAQ-R) of the respondents studied, *N* = 200

Variable	Range	Mean ± SD
NAQ-R Total	1–5	2.17 ± 1
Person-oriented bullying	1–5	2.01 ± 0.97
Work-related bullying	1–5	2.37 ± 1.17
Physical intimidating forms of bullying	1–5	2.35 ± 1.25

Table 4 presents the internal consistency of the negative act questionnaire in the current study, the Cronbach’s Alpha for the total NAQ-R, person-oriented bullying, work-related bullying and physical intimidating forms of bullying were 0.957, 0.923, 0.908, and 0.845 respectively. These results indicate excellent reliability for the total NAQ-R, the same for person-oriented and work-related bullying subscales, and good for the physical intimidating forms of bullying subscales.

**Table 4:** Reliability of the Negative Acts Questionnaire-Revised (NAQ-R)

Scale	Number of items	Cronbach’s alpha
NAQ-R Total	22	0.957
Person-Oriented Bullying	12	0.923
Work-Related Bullying	7	0.908
Physical Intimidating Forms of Bullying	3	0.845

Table 5 presents the relationships between the three dimensions of bullying, as measured by the Negative Acts Questionnaire-Revised (NAQ-R), and demographic and work-related data. A statistically significant association was observed between bullying experience and nurse interns’ age across age groups (*p* < 0.001, *p* < 0.001, *p* < 0.002), with scores increasing with age and peaking at 26–29 years. Concerning gender, it is also found that males report slightly higher bullying scores across all types than females; however, a non-statistically significant association was observed. Also, there was a non-statistically significant association between experience of bullying and the work unit, and marital status.

**Table 5:** Relationship between the three dimensions of bullying measured by Negative Acts Questionnaire-Revised (NAQ-R), and demographic, work-related data

Variable	Categories	*n*	Person-oriented bullying score	Work-related bullying score	Physical intimidating forms of bullying score
Age Group	22 Yrs	86	1.85 ± 0.71	1.99 ± 0.91	2.10 ± 1.15
	23 Yrs	42	1.96 ± 1.05	2.30 ± 1.07	2.13 ± 1.15
	24 Yrs	22	1.95 ± 1.07	2.74 ± 1.29	2.67 ± 1.42
	25 Yrs	18	2.25 ± 1.01	2.95 ± 1.41	2.78 ± 1.04
	26–29 Yrs	14	3.36 ± 1.33	3.49 ± 1.39	3.38 ± 1.57
	30 + Yrs	18	1.72 ± 0.58	2.44 ± 1.09	2.44 ± 1.18
	ANOVA (p)		F = 7.46 (*p* < 0.001)	F = 6.56 (*p* < 0.001)	F = 3.83 (*p* = 0.002)
Gender	Female	160	1.96 ± 0.84	2.32 ± 1.06	2.30 ± 1.17
	Male	40	2.22 ± 1.37	2.59 ± 1.53	2.55 ± 1.52
	t-test (p)		t = 1.53 (*p* = 0.128)	t = 1.3 (*p* = 0.195)	t = 1.13 (*p* = 0.259)
Work Unit	ER ICU	12	2.21 ± 0.68	2.19 ± 0.76	1.78 ± 1.09
	General ICU	170	1.99 ± 0.99	2.34 ± 1.20	2.37 ± 1.23
	Surgical ICU	18	2.13 ± 0.93	2.76 ± 1.01	2.56 ± 1.46
	ANOVA (p)		F = 0.42 (*p* = 0.661)	F = 1.21 (*p* = 0.302)	F = 1.53 (*p* = 0.22)
Marital Status	Single	151	2.04 ± 0.97	2.32 ± 1.15	2.32 ± 1.24
	Married	45	1.99 ± 1.02	2.63 ± 1.21	2.53 ± 1.32
	Others	4	1.33 ± 0.29	1.29 ± 0.33	1.50 ± 0.58
	ANOVA (p)		F = 1.04 (*p* = 0.355)	F = 2.98 (*p* = 0.053)	F = 1.42 (*p* = 0.245)

**The findings in** Table 6** revealed four preponderant themes**:

**Table 6:** Analysis of intern nurses’ perspectives on perceptions of bullying, emotional reactions to bullying, coping strategies, and their own messages and suggestions to prevent bullying

Theme	Subthemes
1.Professional and social vulnerability to workplace bullying	1.1: Professional inexperience 1.2: Limited hands-on practice and lack of confidence 1.3: Environmental unfamiliarity 1.4: Discrimination related to gender and level of education
2.The emotional reactions to workplace bullying and its effect on intern nurses’ professional conduct.	2.1: Fear of reporting bullying 2.2. Uncertainty about identifying bullying 2.3: Disruption of professional identity
3.Coping with workplace bullying	3.1. Normalization of bullying 3.2. Internalization of experience 3.3: Avoidance behaviors 3.4. Assertive copying
4. Explicit messages and suggestions	4.1: Calling for education and awareness on bullying 4.2: Establishing institutional anti-bullying policies and accountability 4.3: Organizational culture change 3.5. Self-developed resilience approaches

## Professional and social vulnerability to workplace bullying

### Professional inexperience

The vulnerability to workplace bullying is not merely an individual characteristic but rather emerges from professional and social factors. Professional inexperience emerged as a significant vulnerability factor among participants. Newly qualified professionals described feelings particularly susceptible to bullying behaviors due to their limited professional history and unfamiliarity with workplace dynamics.

*I felt like I had a target on my back because I was new. Every time I asked a question*,* they would roll their eyes and say*,* ‘Didn’t they teach you anything in school?’ (P2*).

The inexperienced participants often struggled to distinguish between legitimate professional guidance and inappropriate behavior.

### Limited hands-on practice and lack of confidence

Limited hands-on practice highlighted how insufficient practical experience created opportunities for workplace bullying. Participants described situations where their limited practical skills were exploited by colleagues who used this vulnerability to undermine their confidence and professional standing.

*When I was learning a procedure*,* my supervisor would stand behind me and loudly criticize every move I made in front of other staff. She’d say things like ‘Are you sure you graduated?’ when I hesitated (P1)*.

Lack of confidence emerged as both a precursor to and consequence of workplace bullying vulnerability. Participants consistently identified low self-confidence as a factor that made them attractive targets for bullies.

*I was already nervous about being in a new environment*,* and they could sense my insecurity. It was like they smelled fear and decided to make it worse* (P 4).

### Environmental unfamiliarity

An unfamiliar environment represented a significant vulnerability factor, particularly for intern nurses in their transition between different workplace cultures. Participants reported that a lack of understanding of informal rules, organizational culture, and interpersonal dynamics left them exposed to bullying behaviors.

*I didn’t know the unwritten rules of the unit. When I made innocent mistakes about procedures or protocols*,* they treated me like I was incompetent rather than just unfamiliar with their way of doing things* (P 25).

### Discrimination related to gender and level of education

Gender bias emerged as a pervasive factor contributing to workplace bullying vulnerability. Participants identified various forms of gender-based discrimination that created or exacerbated their susceptibility to bullying behaviors. The female participant reported experiencing bullying that was specifically tied to gender stereotypes and expectations. This included questioning their competence in traditionally female-dominated areas. The data showed that gender bias often intersects with other vulnerability factors, creating compound disadvantages.

*They would constantly question my physical strength and ability to handle certain tasks*,* even though I was perfectly capable. It was always ‘Are you sure you can lift that?’ or ‘Maybe you should get one of the guys to help you’* (P 23).

Male participants also described experiences of gender bias, particularly when their behavior or characteristics did not conform to traditional masculine stereotypes.

*Because I was more soft-spoken and preferred to avoid conflict*,* they questioned my ability to handle pressure. They would say things like ‘This job isn’t for people who can’t be tough (P3).*

Discrimination based on the level of education revealed how participants’ educational background became a source of vulnerability to workplace bullying and was used to discredit their practical abilities and suggest arrogance instead of being recognized as a strength. This example highlights how bullying can be subtle, personal, and tied to professional identity, especially in environments where experience is valued over formal education. A participant said:

*They used my academic background against me*,* saying I was ‘all theory and no practice’ and that I thought I was better than everyone else just because I had good grades* (P5).

Bullying in this condition may reflect underlying jealousy or insecurity and serve to isolate the individual, undermining their confidence and sense of belonging.

## The emotional reactions to workplace bullying and its effect on intern nurses’ professional conduct

### Fear of reporting bullying

Participants feared professional consequences, such as poor evaluations, damaged reputations, or being labeled “difficult,” if they reported bullying.

*If you complain*,* they’ll say you’re not resilient. And those sticks—it follows you in your career* (P 12).

Another participant emphasized being quiet and avoiding reporting bullying.

*You’re scared it’ll get worse if they find out you said something. Sometimes it’s safer to just keep quiet (P14).*

This fear was intensified by a perceived lack of confidentiality in reporting systems and a belief that leadership often sided with more senior staff.

### Uncertainty about identifying bullying 

Interns expressed uncertainty about what behaviors constituted bullying, especially when feedback was delivered aggressively or in front of others.

*“Sometimes I wonder—is this bullying or just feedback? The tone is harsh*,* but maybe I needed to hear it?” (P20).* Other participants demonstrated that a lack of clear definitions and training about bullying creates a grey area that allows harmful behaviors to persist. *“Nobody defines what bullying is. You’re just told to be strong*,* so when something feels off*,* you’re not sure if you’re overreacting” (P22).*

This ambiguity contributed to silence and underreporting. Interns hesitated to label their experiences as bullying without clear institutional guidance or peer validation.

### Disruption of professional identity

Many participants described how bullying interfered with their developing professional identity. *“I used to see myself as someone who would champion patient advocacy.*

*Now I’m afraid to speak up about anything*,* even when I see something concerning (P8).*

Participants consistently reported a range of immediate emotional reactions when experiencing bullying in professional settings.

*Being criticized so harshly in front of patients made me feel incredibly small. The shame was overwhelming*,* I questioned if I belonged in this profession at all (P 13).*

## Coping with workplace bullying

The coping strategies described by participants ranged from passive reactions such as normalization, internalization, and avoidance behavior to assertive coping.

### Normalization of bullying

Some participants adopted a strategy of normalizing bullying behaviors by viewing them as an inherent aspect of workplace culture. This cognitive reframing allowed interns to rationalize their experiences and reduce the immediate emotional impact of bullying incidents.

*I just told myself that this is how things are in healthcare. Everyone who came before me went through the same thing*,* so I needed to toughen up and accept that it’s part of paying your dues (P 6).*

*It’s almost like a rite of passage. Everyone says*,* ‘we went through it too*,*’ so you’re expected to just deal with it (P 7)*.

Another participant describes it as a way of tough love.

*It happens so often that people don’t even call it bullying. They say things like ‘tough love’ or ‘building resilience* (*P 10).*

This normalization led many interns to question whether their experiences constituted bullying or were simply part of the learning process. Participants described how this normalization process helped them maintain their commitment to completing their internship despite adverse experiences. However, the data revealed that this strategy often came at a high psychological cost. As one student reflected:

*I kept telling myself it was normal*,* but deep down I knew it wasn’t right. I was just trying to survive each day by pretending it was okay.* (P11).

### Internalization of experience

The internalization strategy often led to increased self-doubt and a decline in self-worth among participants. Intern nurses described engaging in extensive self-criticism and constantly questioning their abilities and decisions.

*I kept thinking that if I just worked harder*,* studied more*,* or stayed quieter*,* maybe they would stop picking on me. I thought it was my fault for not being good enough (P16).*

### Avoidance behaviors

Some participants employed avoidance behaviors to minimize their exposure to bullying. This approach involved either physically or emotionally distancing themselves from situations or individuals associated with bullying. A participant described:

*I learned to map out where the bullies would be and planned my routes to avoid them. I would take longer paths just to avoid walking past their station* (P 21).

Some participants resorted to missing shifts when the stress and anxiety of facing workplace bullying became overwhelming.

*I started finding excuses not to show up on days when I knew certain staff members would be working. I told myself I was protecting my mental health*,* but I knew I was also hurting my education (P15).*

Some participants asked for a transfer to a different unit, which emerged as a strategy employed by nurses who felt unable to cope with the bullying in their assigned placement. This approach represented an attempt to escape the immediate bullying situation while maintaining their educational progress.

*I told my academic advisor that I wasn’t getting good learning experiences in my current placement*,* but really I just needed to get away from the people who were making my life miserable. (P18).*

### Assertive coping

Some participants adopted more proactive approaches to address workplace bullying. These involved direct action to either prevent future incidents or address ongoing problems through skill development or direct confrontation. Participants tended to identify their knowledge or skill gaps and actively sought additional learning opportunities to reduce their vulnerability to bullying. This strategy was based on the belief that improved competence would make them less attractive targets for criticism and harassment.

*I realized that some of the bullying was happening because I lacked certain skills*,* so I started asking for extra practice time and seeking out additional training opportunities (P 9).*

The most direct strategy was to confront the bully and explicitly ask them to stop their behavior. This approach required significant courage and was often attempted only after other strategies had failed or when the bullying became intolerable. Some found that addressing the behavior directly led to positive changes in their relationship with the bully.

*I waited until we were alone and said*,* ‘I feel like you have been singling me out unfairly*,* and I would appreciate it if we could find a more constructive way to work together (P29).*

## Explicit messages and suggestions

### Calling for education and awareness on bullying

Intern nurses’ own messages and suggestions consistently emphasized the need for nursing education programs to better prepare students for the realities of workplace bullying.

*Nursing schools need to stop pretending bullying doesn’t exist. We need specific training on how to respond professionally to lateral violence. I wish my program had included role-playing exercises on handling difficult colleagues and explicit strategies for maintaining confidence when being criticized unfairly. Going in prepared would have made such a difference (P 26).*

These recommendations highlighted a perceived gap between nursing education and workplace realities, leaving many novice interns feeling unprepared for the interpersonal challenges they faced.

### Establishing institutional anti-bullying policies and accountability

A significant number of participants emphasized the need for explicit anti-bullying policies with genuine accountability measures, and to clearly announce these policies in the health care setting.

*Hospitals need zero-tolerance policies for bullying that are actually enforced, not just mentioned during orientation and then ignored. There should be clear, confidential reporting systems and mandatory investigations. When I finally reported what was happening, I was told to ‘work it out’ with the person bullying me, which only made things worse (P 27)*.

### Organizational culture change

Participants offered thoughtful suggestions for broader change initiatives within the nursing culture.

*"Hospitals should implement regular ‘culture rounds’ where executives and nurse leaders round on units specifically to ask about team dynamics and psychological safety. Make workplace culture as important a metric as patient satisfaction or clinical outcomes. Measure it, track it, and tie leadership evaluations to it" (P19).* These suggestions demonstrated participants’ understanding of how organizational priorities and reward systems shape workplace culture.

Participants also directly addressed experienced nurses, appealing to them to become active allies.

*To experienced nurses: You have so much power to change this culture. When you see a colleague being harsh to a new graduate*,* please say something. Your silence is interpreted as agreement. You don’t have to make a scene—just a simple ‘that approach isn’t helpful’ or taking the intern aside afterward to validate that the interaction wasn’t appropriate can make all the difference (P 17).*

These direct appeals revealed participants’ awareness that cultural change requires active participation from those with established positions in the nursing hierarchy.

### Self-developed resilience approaches

Although self-care alone is not enough to resolve bullying problems and systemic change is essential, participants emphasized the importance of building personal resilience as a supportive measure for new nurses navigating challenging work environments.

*… new nurses also need to develop their own resilience toolkits. I’d recommend that every intern finds a mentor outside their unit*,* keeps a reflective journal*,* and establish firm boundaries between work and personal life. Self-care isn’t the solution to bullying*,* but it’s necessary while we work on changing the culture (P 28).*

## Discussion

Workplace bullying in the nursing profession encompasses a variety of damaging behaviors, ranging from emotional abuse and harassment to intimidation and physical aggression. The internship period represents a crucial transition from academic study to professional practice, making it a particularly vulnerable time for nursing interns [16, 40]. As a result, this study specifically examines the prevalence of bullying among intern nurses in the ICU.

The study found that more than half of the participants were subjected to bullying, and approximately half witnessed it. This study’s participants reported that they were subjected to bullying due to limited experience, unfamiliarity with patient care routines, unit equipment, institutional policies and procedures, and insufficient competence in carrying out clinical skills. In addition, the hierarchical structures and stress-inducing circumstances in the ICU may induce bullying by other nursing personnel.

Previous studies, in line with this study’s findings, reported that approximately four out of five participants experienced workplace bullying as a victim, a witness, or both, while approximately two out of three reported being bullied as a victim, and approximately four out of five participants [17]. Also, Birks et al. (2024) [18] mentioned in their study that more than a quarter of nursing students reported that they had experienced bullying during clinical placement. Zhang et al. (2024) [19] found that senior nursing students had experienced various types of bullying. Also, Fernández-Gutiérrez et al. (2024) [20] reported that the majority of participants in their study were victims of bullying. Pan et al. (2024) [21] found that bullying occurred to 65.7% of fourth-year bachelor’s nursing students. Moreover, a qualitative study conducted in Ghana revealed that nursing interns had experienced bullying practices such as shouting, isolation, humiliation, and being assigned tasks below their competency level [22]. 

As concern types of bullying, it was observed from this study findings that work-related bullying as measured by negative act questionnaire (NAQ-R) in form of by high reports of unmanageable workloads, being ordered to do work below competence level, and having one’s opinions ignored and physically intimidating behaviors (excessive monitoring of work, persistent criticism, and repeated reminders of errors) are slightly more reported than personal attacks (being ignored or excluded, being humiliated or ridiculed in connection with work, and being subjected to practical jokes). Birks et al. (2024) [18] reported similar findings, while near findings were reported by others (2024). They mentioned that nursing interns in Sri Lanka reported the most common forms of bullying they experienced were verbal and non-verbal; however, this study focused on the nursing interns’ experience of bullying during clinical placement. A similar finding to this study using NAQ-R was reported by Serafin et al. (2019) [23], who found that work-related bullying was the most prevalent type among nurses working in Polish healthcare facilities, with items such as unmanageable workload and being ordered to do work below competence level being frequently reported.

Regarding the association between bullying experience and demographic and work-related data, this study’s findings revealed a significant association between bullying experience and intern nurses’ age across age groups, with the scores increasing with age. Concerning gender, it is also found that males report slightly higher bullying scores across all types than females. Also, there was a non-statistically significant association between the experience of bullying in the work unit and marital status. These results may be explained by the fact that nursing is a predominantly female profession, which can lead male interns to face difficulties with role acceptance and possibly be expected to take on more responsibilities than their female counterparts.

Additionally, older intern nurses may have more maturity, which could make them more perceptive of and affected by inappropriate behaviors directed toward them. This, in turn, may lead to greater awareness of and reporting of bullying. On the other hand, the absence of significant associations between bullying experiences and factors such as work unit or marital status suggests that bullying is a pervasive issue within healthcare settings, not confined to specific units or influenced by intern nurses’ personal circumstances. Similar results were reported by Ali et al. (2022), who identified significant relationships between intern nurses’ bullying experiences and variables such as age, social status, and educational level prior to enrolling in the faculty.

### Professional and social vulnerability to workplace bullying

Professional inexperience emerges as a critical vulnerability factor that predisposes newly qualified nurse interns to workplace bullying. The elevated levels of workplace bullying among intern nurses presented in the quantitative findings are explained by the qualitative theme of **professional and social vulnerability**. Intern nurses’ professional inexperience limited hands-on practice, and lack of confidence increased their vulnerability to criticism and unrealistic expectations, particularly in high-acuity ICU environments. Qualitative accounts further showed that unfamiliarity with unit routines and technology often resulted in task overload and fault-finding, which aligns with the higher work-related bullying scores observed quantitatively. In addition, experiences of discrimination related to gender and level of education highlight how hierarchical and social power imbalances exacerbate bullying behaviors, reinforcing the vulnerability of intern nurses within clinical teams.

Young and inexperienced nurse interns demonstrate higher rates of workplace bullying exposure. In congruence with Chatziioannidis et al. (2018) [24], who supports our findings about the heightened susceptibility of new professionals. The vulnerability of inexperienced professionals stems from multiple interconnected factors. New graduates often lack the organizational knowledge and social capital needed to navigate complex workplace dynamics, leaving them vulnerable to aggressive behavior. Their unfamiliarity with professional boundaries creates ambiguity in distinguishing between legitimate feedback and inappropriate conduct.

The vulnerability created by insufficient practical experience represents a critical pathway through which workplace bullying manifests, particularly in professional environments where competency is directly observable. Limited hands-on practice creates multiple opportunities for colleagues to exploit these skill deficits, recognizing and targeting them as points of professional weakness. It aligns with Balducci et al. (2020) [25] who found that the visible nature of practical skills creates a particularly exposed position for new professionals, as their competency gaps are immediately apparent to colleagues and supervisors.

The identification of low self-confidence as both a precursor to and consequence of workplace bullying reveals the cyclical nature of bullying vulnerability. This dual relationship creates a self-perpetuating cycle where initial insecurity attracts bullying behaviors, which subsequently further confidence, making individuals increasingly susceptible to continued victimization. The Findings are in congruence with Mehmood et al. (2024) [26] who strongly support that bullies can sense insecurity and exploit it strategically.

The transition between different workplace environments creates a particularly precarious situation in which individuals must navigate complex social and professional landscapes without a clear understanding of implicit expectations and behavioral norms. The distinction between incompetence and unfamiliarity is crucial yet often deliberately obscured by workplace bullies. Unfamiliarity with “unwritten rules” creates a double vulnerability: newcomers are expected to intuitively understand implicit organizational culture while simultaneously being denied the supportive socialization processes that would facilitate this understanding. This finding is in line with Quinn et al. (2024) [27] who found that in a poorly organized work environment, employees experience high levels of stress and frustration, which may lead them to be involved in interpersonal conflicts, with some of these conflicts spiraling and evolving into bullying situations.

Gender bias emerges as a pervasive vulnerability factor that operates through the enforcement of traditional gender stereotypes, creating distinct pathways to workplace bullying for both male and female workers. This bias functions as an intersectional vulnerability that compounds other risk factors, creating complex layers of disadvantage that perpetrators exploit systematically. For female participants, gender bias manifests through systematic questioning of competence in traditionally male-dominated areas. The persistent challenges to physical capability go beyond a simple concern for safety. They constitute deliberate undermining of professional competence based on gender assumptions. This finding is supported by Striebing (2022) [28], who found that competency challenges in male-dominated tasks become tools of professional diminishment.

Male participants face a different but equally damaging form of gender bias when their behavior deviates from masculine stereotypes. The criticism of being soft-spoken and the questioning of one’s ability to handle pressure reveal how non-conformity to aggressive masculine norms becomes a vulnerability. In alignment with Rosander et al. (2020) [29] who demonstrate that men, when in a gender minority, face increased risk of bullying, particularly for person-related negative acts, suggesting that men who don’t conform to dominant workplace masculinity face targeted harassment.

Educational background emerges as a complex vulnerability factor that creates distinct pathways to workplace bullying depending on the organizational context and the perceived status of one’s academic credentials. The double-edged nature of educational vulnerability creates a particularly insidious situation where individuals cannot escape discrimination regardless of their academic background. Those with lower educational credentials face challenges related to perceived incompetence, while those with higher credentials encounter resentment and anti-intellectual bias. In line with Zou et al. (2024) [7] who concluded that implementing specialized education during the internship phase offers valuable insights for contemplating how to reform specialized teaching to meet the evolving demands of nursing in the new era.

### The emotional reactions to workplace bullying and its effect on intern students’ professional conduct

The participants’ fears of poor evaluations, damaged reputations, and being labeled as difficult reflect a rational assessment of the professional risks associated with reporting workplace bullying in healthcare settings. This rational calculation is grounded in rational choice theory, which suggests that individuals make decisions by systematically weighing costs and benefits to maximize their personal advantage. When participants engage in cost-benefit analysis, they recognize that the potential costs of reporting, including career damage, professional stigmatization, and long-term reputation harm, consistently outweigh the uncertain benefits of intervention. Al Omar et al. (2019) [30] support our findings as their study reported that healthcare workers systematically evaluated the potential negative consequences of speaking up, including damage to relationships, reputation, and career prospects.

The participants’ uncertainty about what constitutes bullying reflects a critical gap in organizational clarity, with profound implications for reporting and intervention. A study by Blomberg et al. (2024) [31] found that the lack of clear definitions creates a gray zone where harmful behaviors persist because victims are uncertain whether their experiences meet the threshold for formal complaint. The ambiguity surrounding what constitutes workplace bullying makes it difficult for employees to identify and report such behaviors, as they may question whether their experiences qualify as bullying or are simply part of normal workplace interactions.

The confusion between aggressive feedback and bullying is particularly problematic in healthcare settings where direct, sometimes urgent communication is necessary for patient safety. These findings are in line with Koh (2016) [32], who demonstrates that this definitional ambiguity is often exploited by perpetrators who can frame their behavior as tough love or necessary professional development, making it difficult for targets to challenge the behavior without appearing weak or oversensitive. The healthcare context amplifies this problem because aggressive communication can be rationalized as patient advocacy or clinical urgency, creating additional layers of justification for potentially harmful behaviors.

The interference of bullying with professional identity formation represents one of the most serious long-term consequences for intern nurses. Professional identity formation is recognized as an integral part of professional development, occurring dynamically and longitudinally through immersion in the socialization process. However, our participants’ experiences reveal how bullying fundamentally disrupts this critical developmental process. This aligns with Yang et al., (2023) [33] who found that workplace violence can affect patient safety through decreasing professional identity and increasing professional burnout among intern nurses.

### Coping with workplace bullying

Some participants in the study coped with bullying by normalizing it as a routine aspect of healthcare culture. This approach allowed them to reduce the emotional impact of bullying by viewing it as an expected rite of passage. A study by van Heugten et al. (2018) [34] who show that such normalization is common in healthcare environments, where hierarchical structures and cultural silence often lead individuals to accept bullying rather than challenge it. While this strategy may offer short-term emotional relief, it risks perpetuating a toxic culture and discouraging systemic change.

The internalization of bullying experiences often led to heightened self-doubt and reduced self-worth among intern nurses. Participants reported engaging in persistent self-criticism, frequently questioning their clinical abilities and decision-making skills. These findings are in line with Yosep et al. (2022) [44] who show that individuals exposed to repeated bullying tend to internalize negative feedback, leading to psychological distress and a diminished sense of professional competence.

Participants often coped with bullying by employing avoidance, which involved both physical and emotional distancing. These strategies, though intended to minimize harm, placed a significant cognitive and emotional burden on intern nurses. Consistent with Van Den Brande et al. (2017) [35] who found that such behaviors reflect a survival-oriented response that may offer temporary relief but ultimately contribute to chronic stress, reduced psychological safety, and disengagement from the learning environment.

Some participants resorted to missing shifts as a coping mechanism when the stress and anxiety of workplace bullying became unmanageable. This form of avoidance reflected the intensity of their emotional distress and a perceived lack of safer alternatives. Choosing absence over confrontation highlights how bullying can severely impact intern nurses’ well-being and clinical engagement. Similar findings in a recent study by Johnson et al. (2024) [36], who found that absenteeism is often a last resort, signaling environments where psychological safety is compromised and institutional support is lacking.

Some participants requested transfers to different units as a coping strategy when bullying in their assigned placements became intolerable. This approach allowed them to escape the immediate source of distress while continuing their clinical education. It reflects self-advocacy and a desire to preserve both mental health and academic progress. However, this strategy also highlights institutional gaps in addressing bullying directly, as intern nurses often felt that removal from the environment rather than resolution of the issue was the only viable solution. These findings are in congruence with Amoo et al. (2021) [22] who found that transfers are used to avoid, rather than confront, toxic workplace cultures.

Assertive coping strategies emerged as proactive approaches used by some participants to address workplace bullying. Rather than avoiding or internalizing the experience, these intern nurses equip themselves with knowledge and skills to master their competencies and increase their confidence in their ability to provide patient care, believing that improving their clinical skills would reduce their vulnerability to criticism and mistreatment. By actively seeking additional learning opportunities and directly confronting challenges, they aimed to shift the power dynamic and assert greater control over their experiences. These findings are in line with Ribeiro & Sani (2024) [37] who suggest that assertive coping can enhance resilience and promote a stronger sense of agency, though its effectiveness often depends on the broader supportiveness of the clinical environment.

The most straightforward way to cope was to directly address the bully and ask them to stop their behavior. This method required significant courage and was usually attempted as a final option, or when the bullying reached an intolerable level”. For some participants, directly addressing the issue increased the likelihood of further incidents. The findings are in congruence with Van Den Brande et al. (2017) [35] who concluded that assertive confrontation, while challenging, can be effective in disrupting bullying dynamics when supported by a respectful environment.

### Intern nurses’ own messages and suggestions to prevent workplace bullying

Nurse interns consistently emphasized the urgent need for nursing education programs to address workplace bullying more explicitly. Many called for specific training, such as role-playing difficult interactions and strategies to maintain confidence when unfairly criticized. Incorporating such practical preparation could better support intern nurses’ resilience and professional development. A study conducted by Gillespie et al. (2015) [38] who found that role-play simulations effectively prepared intern nurses to manage bullying behaviors, enhancing both cognitive and emotional responses. Similarly, Yosep et al. (2022) [44] reported that cognitive rehearsal training, which includes role-playing, was well-received by intern nurses and improved their confidence in addressing bullying situations. These interventions demonstrate the value of integrating active learning strategies into nursing curricula to better prepare students for the realities of workplace bullying.

Many participants stressed the importance of having explicit, well-enforced anti-bullying policies in healthcare settings. They called for zero-tolerance approaches that go beyond mere mention during orientation, emphasizing the need for clear, confidential reporting systems and mandatory investigations. Such measures are crucial to ensure accountability and protect staff from bullying, as research shows that effective policy enforcement is key to reducing workplace harassment and fostering a safer, more respectful environment. In line with Abou El Yazied et al. (2024) [8] who concluded that implementing bullying prevention guidelines for intern nurses was effective in improving their level of assertiveness in clinical practice during the internship year.

Participants proposed meaningful strategies to foster cultural change in nursing, emphasizing the need to prioritize psychological safety alongside clinical outcomes. Participants suggested implementing regular culture rounds in which leaders actively assess team dynamics and the workplace climate. This reflects an awareness of how organizational priorities and reward systems influence behavior and norms. This finding is in line with Sexton et al. (2021) [39] who highlight that leadership engagement and visible commitment to staff well-being are key drivers of a healthy work environment.

While most participants emphasized the need for systemic interventions to address workplace bullying, many also recognized the importance of developing personal resilience strategies. This dual approach reflects an understanding that while organizational change is essential, individual coping tools such as mentorship, reflective journaling, and setting personal boundaries are necessary for navigating current challenges. A study by Foster et al. (2018) [10] who support this perspective, showing that resilience-building can buffer the psychological impact of bullying and enhance intern nurses’ capacity to cope during periods of cultural transformation. Together, these strategies and more supportive work environments.

Participants made direct appeals to experienced nurses, urging them to become active allies in addressing workplace bullying. These appeals demonstrated a clear understanding that meaningful cultural change requires the involvement of those with influence and authority within the nursing hierarchy. By encouraging senior nurses to speak up, model respectful behavior, and support junior staff, participants highlighted the potential for allyship to disrupt harmful norms and foster safer, more inclusive environments. This finding is supported by Navarro et al. (2023) [45] who emphasize that leadership and peer advocacy are critical in shaping workplace culture and empowering newer intern nurses.

## Conclusion and recommendations

These findings demonstrate a significant prevalence of bullying in the workplace, based on both firsthand accounts and observed instances. The most prevalent types of bullying were found to be work-related and physically intimidating, highlighting the significance of putting in place focused interventions and institutional policies to deal with and stop these kinds of behaviors, especially in clinical settings. The results also examine the elements that make intern nurses more susceptible to bullying transitions. The study also highlights the coping strategies used by interns in reaction to bullying and provides important messages meant to stop this kind of abuse. Further research should concentrate on the bullying that critical care nurses encounter at various phases of their careers, particularly those who have worked in the field for several years.

To help intern nurses cope with difficult clinical experiences, it is necessary to increase their awareness of bullying and teach coping mechanisms during pre-internship training. Additionally, mentorship programs established by faculty stakeholders must be implemented to provide an adequate support system. Additionally, interns should be made aware of a clear, private procedure for reporting bullying incidents so they can do so without fear of reprisals. Any reported incidents should be handled immediately, and the results should be openly communicated to nursing interns.

## Limitation of the study

The study recognizes that there are various limitations that could limit the generalizability of the results. First, the research focused on intern nurses working specifically in intensive care units, and hence, the results could not be generalizable to other fields for intern nurses. Moreover, workplace bullying depends on various individual, social, and socio-cultural factors. Hence, there could be differences in results for different nations and socio-cultural settings. To overcome such limitations, there was an effort to increase diversity through the inclusion of participants with different individual and socio-cultural attributes.

## Data Availability

All data generated or analyzed during this study are available from the corresponding author upon request.
